# Case report: Cochlear implantation for deafness caused by endolymphatic sac tumors in patients with von Hippel–Lindau syndrome

**DOI:** 10.3389/fonc.2024.1443370

**Published:** 2024-11-13

**Authors:** Oskar Rosiak, Nikodem Pietrzak, Katarzyna Jankowska, Andrzej Kucharski, Wiesław Konopka, Marcin Szymański

**Affiliations:** ^1^ Department of Otolaryngology, Polish Mother’s Memorial Hospital Research Institute, Łódź, Poland; ^2^ Department of Otolaryngology Head and Neck Surgery, Medical University of Lublin, Lublin, Poland

**Keywords:** endolymphatic sac tumor, hearing loss, deafness, von Hippel–Lindau, cochlear implant

## Abstract

**Introduction:**

Endolymphatic sac tumors (ELSTs) are rare neuroectodermal neoplasms that originate in the endolymphatic sac and duct. They exhibit no specific age or gender predilection, although they are more prevalent in patients with von Hippel–Lindau syndrome.

**Material and methods:**

The manuscript preparation adhered to the CARE guidelines for standardizing clinical cases and the PRISMA guidelines for scientific reviews. Three studies that met the inclusion criteria were selected from an analysis of 15 articles, supplemented by two additional studies identified through citation review.

**Results:**

The presented case describes a 16-year-old boy diagnosed with unilateral sensorineural hearing loss secondary to bilateral ELST. Surgical excision of the right ear tumor with simultaneous cochlear implantation was performed, followed by left ear surgery. Hearing restoration was achieved in the implanted right ear, with normal hearing in the left ear, and no postoperative vestibular symptoms were observed.

**Conclusion:**

Prompt surgical intervention remains the cornerstone of ELST treatment. While cochlear implantation is sporadically reported in scientific literature, it offers a potential option for hearing improvement in patients suffering from deafness caused by the disease.

## 1 Introduction

Endolymphatic sac tumor (ELST) is a rare type of neuroectodermal tumor of the endolymphatic sac and duct, showing no predilection for age or sex (1–3). The disease was first described in 1984 when Hassard et al. presented the case of a patient with symptoms of Menier’s disease, in whom an “adenoma of the endolymphatic sac” was discovered during surgery (4). More cases of this type of tumor were reported by Heffner and his team in 1989. They described 20 cases of patients aged 15 to 71 years whose tumors, previously unclassified in the scientific literature, were identified as slow-growing adenocarcinomatous tumors (5). In 1993, Li et al. reclassified earlier descriptions of the disease as intramural pouch tumors (6).

In 1993, Resche et al. noted correlations between the presence of ELST and the von Hippel–Lindau (VHL) syndrome (7). In 2001, Horiguchi et al. published an immunohistochemical study of a patient with an ELST, identifying a molecular association between the tumor and von Hippel–Lindau syndrome (8). As technology has advanced and diagnostic methods have improved, it has been established that endolymphatic sac tumors are more common in patients with VHL syndrome than in the general population. However, they are one of the rarest manifestations of the syndrome. ELSTs in VHL are characterized by more aggressive growth and are typically observed at a younger age than *de novo* cases (9). Current guidelines from the International Consortium for VHL Monitoring Guidelines and the VHL Alliance, 2023, emphasize the importance of thorough history-taking of patients with VHL mutations and regular audiometric testing beginning at age 11 (10).

Endolymphatic sac tumors are characterized by slow growth and local malignancy, with a minimal risk of distant metastasis. They most often develop in the proximal part of the endolymphatic sac (11) and are typically unilateral (12). However, the incidence of bilateral ELSTs in VHL is approximately 1%. Tumor masses usually infiltrate the structures of the inner ear and cause bone erosion of the petrous apex. The growth of the tumor mass toward the cerebellar structures can manifest as cerebellopontine angle syndrome. On histopathological examination, the tumor typically consists of papillary structures and cystic spaces (1, 2, 10, 12–14). One of the first symptoms of ELST can be hearing loss, often sudden, which is related to the vascular nature of the tumor and bleeding into the structures of the inner ear. Other symptoms include tinnitus, balance disorders, dizziness, a feeling of fullness in the ear, and occasionally, facial nerve palsy (1).

Early audiological and vestibular symptoms are often a symptom of small ELST tumors. Audiograms of affected patients frequently show abnormalities, which may include complete or partial hearing loss at various frequencies. Magnetic resonance imaging (MRI) is the most specific and sensitive for diagnosing ELST tumors, while computed tomography (CT) provides information on bone erosion and infiltration of inner-ear structures, which is necessary for surgical planning (2).

The differential diagnosis should exclude inflammatory conditions of the inner ear and tumors of the cerebellopontine angle, such as schwannoma, meningioma, or temporal bone paraganglioma. The currently recommended treatment for ELST is the removal of the tumor with a margin of healthy tissue (15). Two surgical approaches are recommended: a retrolabyrinthine approach for small tumors confined to the temporal bone, which can be extended by a labyrinthectomy in cases where the tumor infiltrates the inner ear, and a retrosigmoid approach for tumors invading the posterior fossa (16). Radiation therapy and radiosurgery are also treatment options for patients with inoperable tumors or residual disease. Due to the frequent recurrence of the disease, patients require long-term follow-up and regular imaging. The disease-free survival rate depends on factors such as the size, location, and timing of surgery, and is approximately 50%–75% (17).

Since the first description of endolymphatic sac tumor, there have been several hundred scientific publications, including nearly 300 studies dedicated to patient case reports. However, reports on bilateral cases of ELST are scarce. In cases of bilateral disease manifesting as profound hearing loss or deafness, cochlear implantation is a therapeutic option for restoring hearing.

The purpose of this study is to present a case of a bilateral endolymphatic sac tumor with unilateral deafness, treated surgically with simultaneous cochlear implantation. Additionally, it aims to review the scientific literature on cochlear implantation in patients with ELST and associated deafness.

## 2 Materials and methods

The “CARE” guidelines were applied to standardize the clinical case description process (18). A literature review was performed using the “PRISMA” criteria (19). A review of the scientific literature was performed on 01 September 2023, using online databases: PUBMED, Embase, and Scopus. “Endolymphatic sac tumor cochlear implant” was formulated as a research question for the review. Eight results from the PUBMED database, five from the SCOPUS database, and 10 from the Embase database. Eight duplicates were removed.

Two authors independently reviewed 15 titles and abstracts selected in the first stage. The inclusion criteria were case reports or clinical case series of patients with ELST undergoing consequent or simultaneous cochlear implantation. Publications in the form of systematic and literature reviews, meta-analyses, or letters to the editor were considered exclusion criteria.

**Figure 1:**
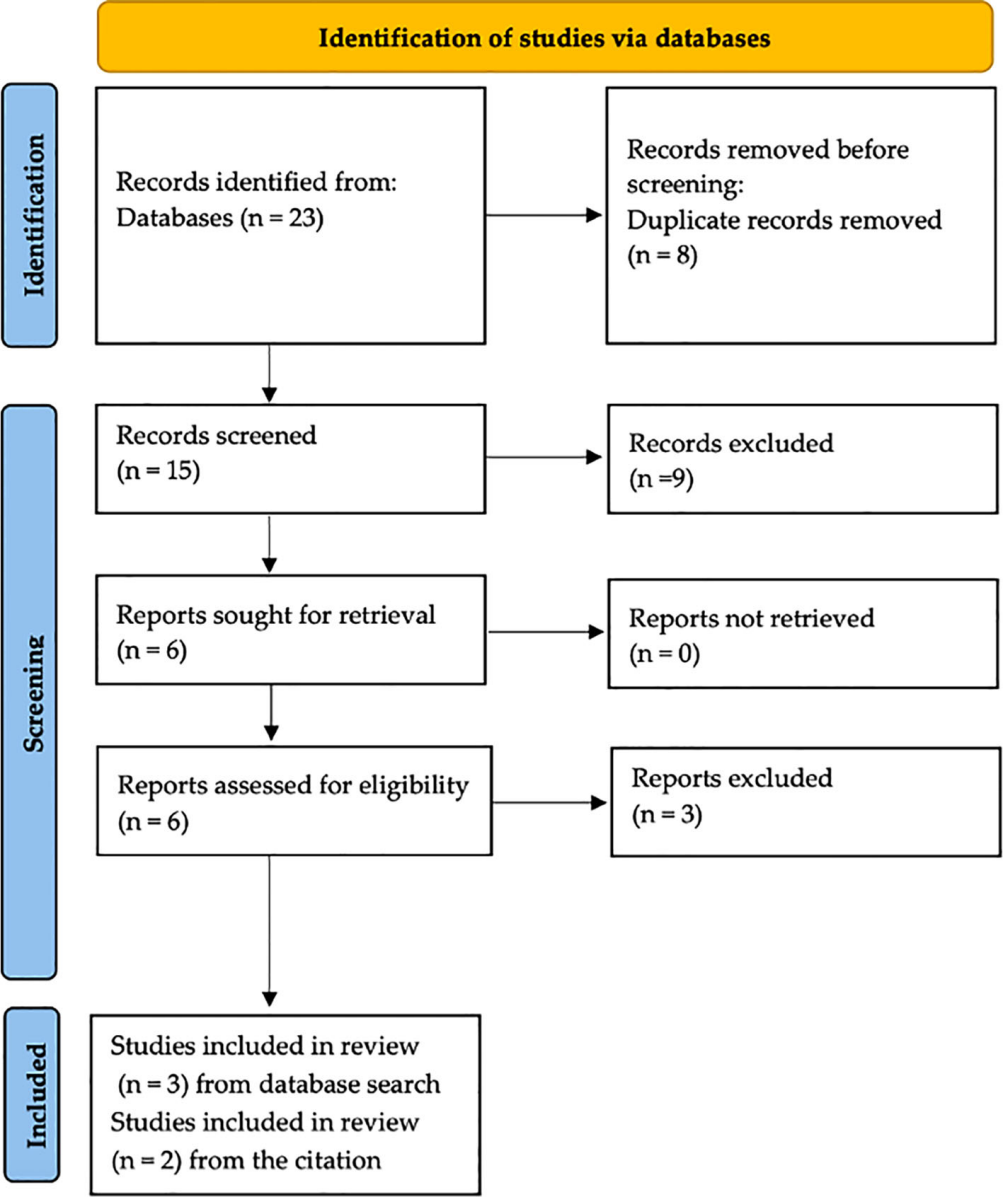
PRISMAFlowchart.

For further review, we included three available studies that met the specified criteria and two additional studies from the citation review (**Figure 1**).

## 3 Case description

The clinical case presented in this study was diagnosed and treated at the Department of Otolaryngology of the Polish Mother’s Memorial Hospital Research Institute in Lodz by one of the authors, as well as at the Department of Otolaryngology and Laryngological Oncology of the Medical University of Lublin. The patients’ medical data were anonymized.

A 16-year-old boy was diagnosed with unilateral sudden sensorineural hearing loss in his right ear at the Department of Otolaryngology of the Polish Mother’s Memorial Hospital Research Institute in Lodz, Poland. The patient developed deafness in the right ear at night, without any preceding symptoms, followed by severe vertigo, the frequency and strength of which decreased in the following months. He presented to the department 4 months after the onset of symptoms. On physical examination, no abnormalities were found, and there was no spontaneous nystagmus; the head impulse test (HIT) was normal. Pure tone audiometry showed deafness in the right ear and normal hearing in the left ear. The videonystagmography (VNG) examination showed peripheral vestibular hypofunction on the right.

### 3.1 Diagnostics

**Figure 2:**
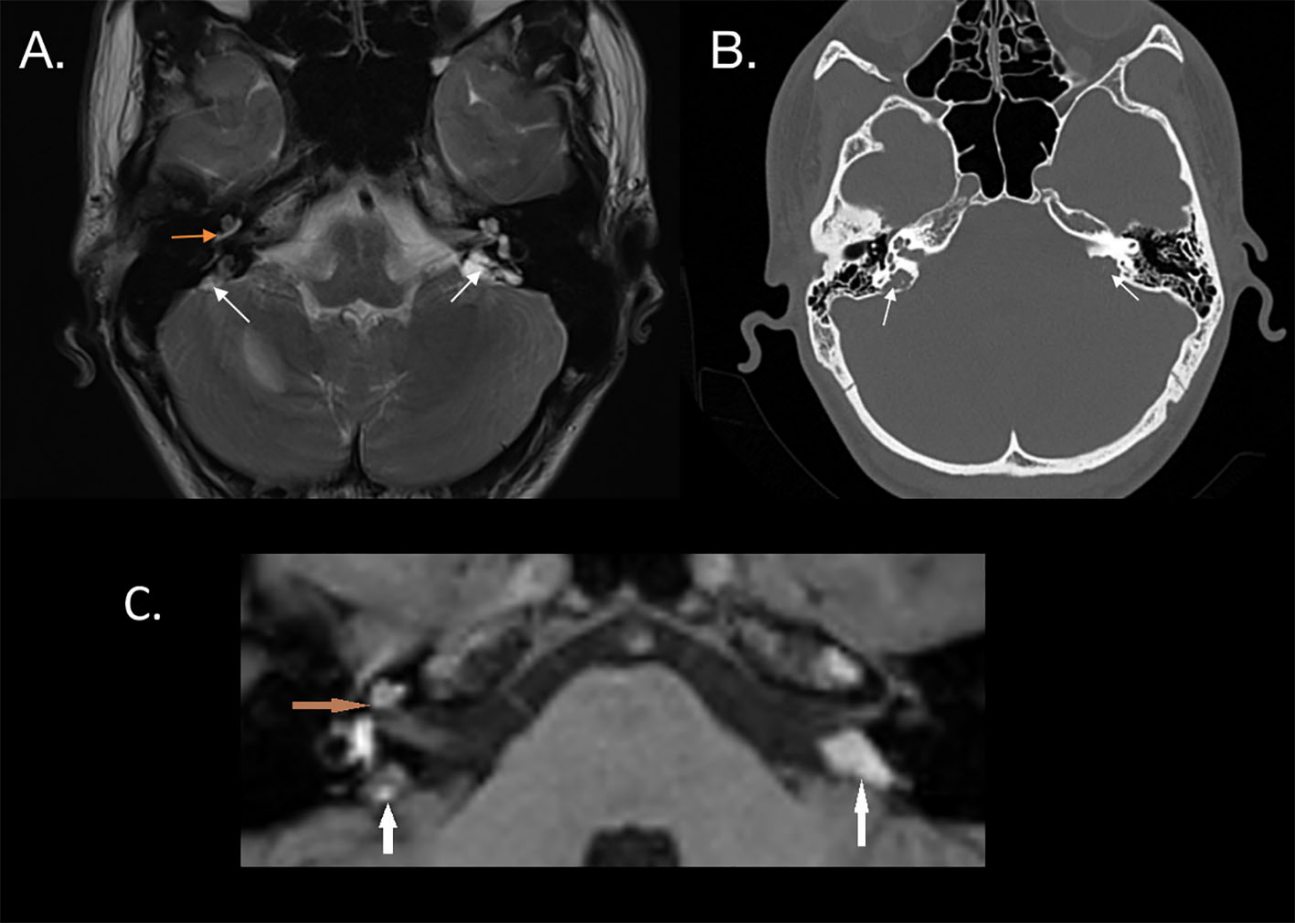
**(A)** MRI T2 sequence of a patient with bilateral endolymphatic sac tumors, indicated by white arrows. Hyperintense fluid in the basal turn of the cochlea, possibly suggesting intracochlear bleeding (orange arrow), is confirmed in the T1 sequence **(C)**. **(B)** CT scan shows a characteristic “moth-eaten” pattern of bone destruction at the location of the endolymphatic sac, involving the vestibular aqueduct (white arrows).

Diagnostic imaging using a T2-weighted sequence revealed a mass of heterogeneous intensity located in the petrous apex was on the right side, along with pathological endolymph signal enhancement in the vestibule and posterior semicircular canal, suggestive of intravestibular blood. On the left side, a larger hyperintense mass was visualized (**Figures 2A, C**). No diffusion restriction was noted on either side, leading to a suspicion of bilateral ELST. The CT scan showed bilateral bone erosion in the area adjacent to the endolymphatic sac and vestibular aqueduct, characterized by a “moth-eaten” pattern and partial destruction of the posterior semicircular canal on the right side (**Figure 2B**).

Given the strong association of the endolymphatic sac tumors with von Hippel–Lindau syndrome, a molecular study was performed on the patient using next-generation sequencing and single amplicon analysis. A heterozygous pathological allele of the VHL gene was identified. Genetic testing of the patient’s parents showed no pathological alleles. The estimated prevalence of this mutation in the Polish population is less than 0.0001.

### 3.2 Treatment

**Figure 3:**
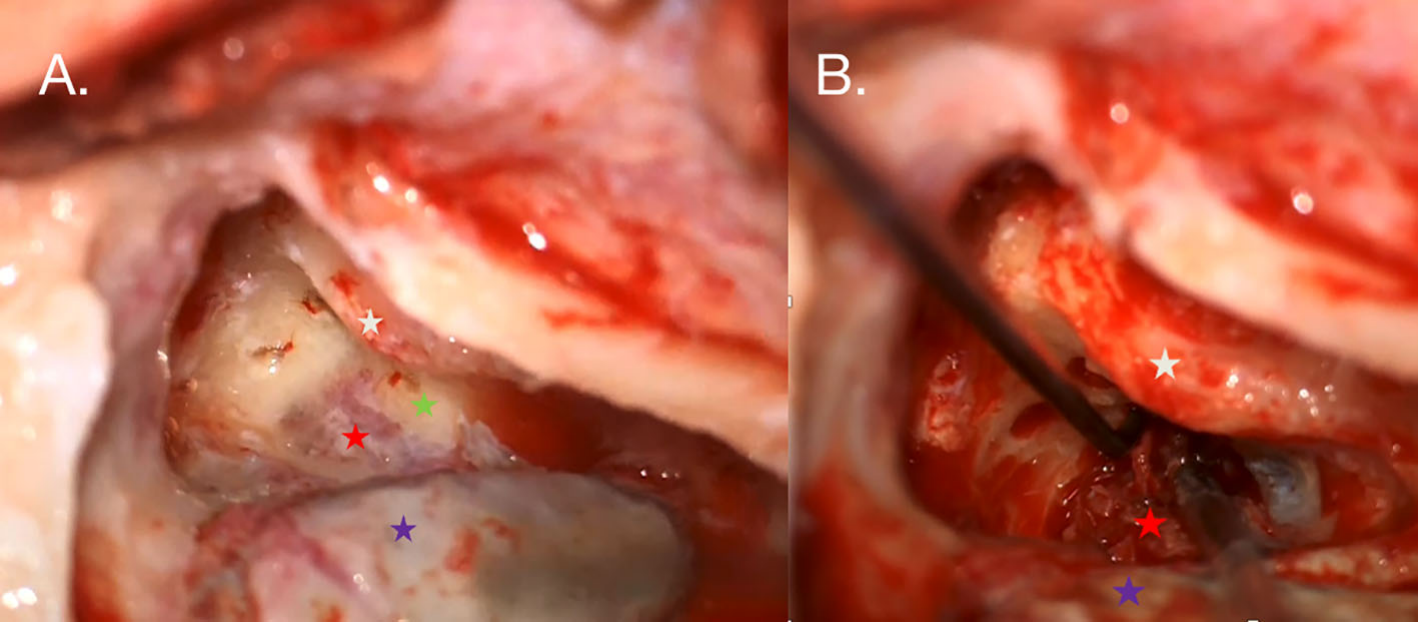
**(A)** Intraoperative photography taken during the right ear procedure. An antromastoidectomy with posterior tympanotomy was performed. The facial nerve (white star) is visualized along the tympanic and mastoid segments. The white arrow indicates the tumor mass (red star) shining under the bone posterior to the posterior semicircular canal (green star), which is partially infiltrated by the tumor. The posterior semicircular and horizontal semicircular canals were drilled out to reveal the extent of the tumor. **(B)** The sigmoid sinus is skeletonized (violet star) and compressed by suction to visualize the highly vascularized tumor.

The right side was chosen for the initial surgery due to the concurrent deafness in that ear. The tumor was removed using a translabyrinthine approach; intraoperatively, the posterior semicircular canal was infiltrated by the tumor mass, and the semicircular canals were drilled to facilitate tumor removal. A minor cerebrospinal fluid leak was observed during surgery (**Figure 3**), and cochlear implantation was performed simultaneously through the round window using the CochlearTM Nucleus^®^ CI612 (Cochlear, Sydney, Australia). After 1 month, the sound processor and cochlear implant were activated. Histopathological examination confirmed ELST.

Due to the high risk of bleeding and hearing loss, surgery on the left ear was scheduled for 1 month later, using a retrolabyrinthine approach. Intraoperatively, no infiltration of the semicircular canals was observed. Most of the suspected tumor mass was confirmed to be hemolyzed blood, while the tumor mass was in fact smaller than that on the right.

**Figure 4:**
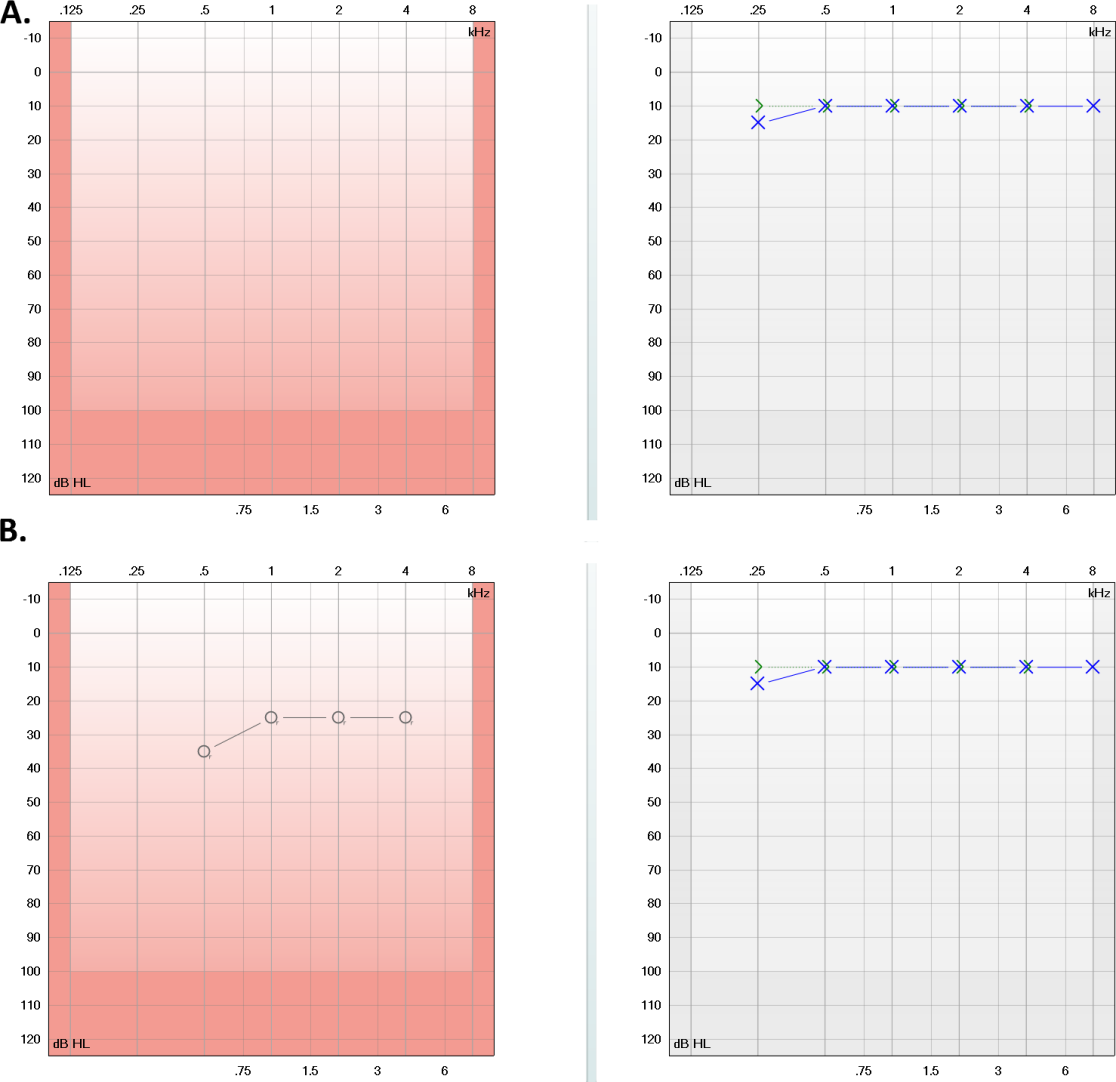
**(A)** Preoperative pure tone audiogram showing deafness in the right ear (red) and normal hearing in the left ear (blue). **(B)** Postoperative audiometry showing free-field results for the right ear and pure tone audiometry for the left ear, indicating normal hearing.

The patient underwent hearing rehabilitation, and the hearing threshold in the right ear was established at 30 dB in free-field audiometry with the sound processor. No postoperative vertigo was observed (**Figure 4**).

## 4 Discussion and literature review

The youngest ELST case included in this review was described by Boccio et al.: a 14-year-old with bilateral ELST tumors (**Table 1**). At presentation, profound hearing loss was already present on both sides. Facial nerve palsy was also observed; however, it is unclear whether the palsy resulted from a prior attempt at tumor removal at a different institution or was a symptom of tumor growth. It is the only reported case where an embolization procedure was performed prior to tumor removal (22). Wick et al. recommend embolization before attempting tumor excision in lesions extending into the posterior fossa, due to the high vascularity of the tumor. Prior embolization is not required in small tumors confined to the temporal bone. Angiography can reveal blood supply from major arteries, including branches of the external carotid artery such as the posterior auricular artery, the occipital artery, and the ascending pharyngeal artery, which can aid in the decision to perform prior embolization (16).

**Table 1:** Summary of literature review on cochlear implantations in patients with hearing loss caused by endolymphatic sac tumors.

Patient age/sex	Author	Symptoms	Diagnostic management	Therapeutic management	Conclusions	Remarks
16/M	Own study	Right sHL, V	Right complete SNHL MRI: bilateral endolymphatic sac lesions CT: bilateral temporal bone lesion “moth-eaten” pattern VHL confirmed Histopathology: bilateral ELST	Total right tumor excision via transmastoid and translabyrinthine approach + cochlear implantation Total left tumor excision transmastoid approach	Hearing is preserved in the left ear and good speech discrimination in the implanted ear Early surgery is recommended to preserve natural hearing; translabyrinthine approach is recommended in the deaf ear	The first reported case of pediatric patient cochlear implantation due to ELST in Poland No clinical manifestation of VHL in medical history or in the family Cochlear™ Nucleus^®^ CI612 Implant and Contour Advance^®^ electrode
36/F	Kveton et al. (20) Poe et al. (21)	Right fHL, V, T (1981); left sHL, V, T (1989)	Bilateral SNHL CT: bilateral lesions ATG: bilateral mildly vascular lesions Histopathology: bilateral papillary adenocarcinoma^*^	Total left tumor excision via transmastoid and translabyrinthine approach + cochlear implantation Total right tumor excision transmastoid approach	Transmastoid labyrinthectomy should be performed if cochlear implantation is considered Auditory performance in these patients can be similar to patients with intact otic capsules	The first reported case of patient cochlear implantation due to ELST Confirmation of VHL disease not mentioned ^*^Diagnosed before tumor reclassification by Li [x] Cochlear™ Nucleus^®^ 22 Implant
14/F	Boccio et al. (22)	Right sHL, T, pain, V + right peripheral facial paralysis	Profound SNHL in the left ear, complete HL in the right ear MRI: bilateral endolymphatic sac lesions Histopathology: ELST ATG: right hypervascular mass VHL confirmed	Prior embolization, total excision of the right mass via petrosectomy and autologous tissue graft^*^ Total left tumor excision via transmastoid approach + cochlear implantation^**^	Early detection of ELST is crucial Management of ELST is complex due to their location and high vascularization—possible hemorrhage High risk of profound hearing loss in bilateral tumors—perform cochlear implantation if possible	^*^Revision surgery after different institution ^**^Nonspecified implant
44/F	Jagannathan et al. (15)	Left HL, V	Severe left SNHL, mild right SNHL progression to severe SNHL in 7 years bilaterally MRI: bilateral ELST CT: bilateral erosion of the posterior petrous temporal bone Histopathology: ELST	Combined transmastoid and retrosigmoid approach with recurrence after 7 years Revision surgery via petrosectomy + cochlear implantation^*^ Total excision of the right tumor^**^	High-res. MRI is important for VHL patients—this factor must be considered before implantation. Recent MR-compatible cochlear implants have addressed this issue For cochlear implantation in ELST patients minimize cochlear trauma in the ELST excision procedure	In a 7-year observation, a progression of HL was noted bilaterally, and a recurrence was observed in the left ear. This is the only reported case in this review with a long-term follow-up ^*^Cochlear™ Nucleus^®^ CI24RE Contour Advance™ ^**^Nonspecified approach
59/M	Holtmann et al. (14)^*^	Left sHL, T^**^	Moderate SNHL in the left ear MRI and CT: left petrous apex lesion involving vestibular aqueduct—retrolabyrinthine and retrocochlear Histopathology: ELST	Transmastoid exploration procedure; subtotal excision of the tumor + CI dummy electrode; adjuvant radiotherapy and gamma-knife surgery cochlear implantation^***^	Early tumor diagnosis and removal are critical In labyrinthectomy surgeries, consider inserting a cochlear implant dummy electrode to prevent cochlear fibrosis and enable 2-stage hearing recovery	^**^The cited publication is a conference poster ^**^No data on the clinical manifestation of VHL disease in patient ^***^No data on cochlear implant

Key for abbreviations: *ELST*, endolymphatic sac tumor; *HL*, hearing loss; *SNHL*, sensorineural hearing loss; *sHL*, sudden hearing loss; *fHL*, fluctuating hearing loss; *HR*, hearing restored; *HP*, hearing preserved; *V*, vertigo; *T*, tinnitus; *BD*, balance disorder; *PTA*, pure tone audiometry; *MRI*, magnetic resonance imaging; *cMRI*, contrast-enhanced MRI; *seq.*, MRI sequence; *high-res.*, high-resolution (MRI); *CT*, computer tomography; *cCT*, contrast-enhanced CT; *ATG*, angiography; *ECA*, external carotid artery; *PET*, positron emission tomography.

In cases where the tumor infiltrates the structures of the inner ear, usually the vestibular aqueduct or posterior semicircular canal, a labyrinthectomy should be considered. This approach enables a more thorough excision of the tumor. Kyeton et al. described a case of a patient with bilateral ELST in which the larger tumor was removed using a translabyrinthine approach, with simultaneous cochlear implantation during the same procedure. The authors point out that when cochlear implantation is considered, a labyrinthectomy can be performed, and the hearing results are similar to those of patients with intact otic capsules. This is also the first reported ELST case in which cochlear implantation was performed; the authors used Cochlear Nucleus 22 Implant (20). VHL disease confirmation was not mentioned in this study, which is not surprising since the histopathological reclassification of ELST by Li et al. was made in 1993 (6). The association of ELST with VHL disease was not proven until 1995 (3).

The follow-up imaging method of choice for suspected recurrence of ELST is MRI. The only reported case of ELST recurrence in this review was presented in 2007 by Jagannathan et al., where ELST recurrence was observed 7 years after the first surgery and correlated with the progression of hearing loss. A revision petrosectomy was performed with simultaneous cochlear implantation.

The authors point out that high-resolution MRI remains a method of choice for detecting ELST recurrence (15). This is especially important for VHL patients, as other hemangioblastomas may occur in the retina or cerebellum. Recent guidelines for patients with confirmed VHL disease recommend a brain MRI every 2 years, starting at age 11, which should continue until age 65 for patients who have never developed a hemangioblastoma. To date, the sensitivity and specificity of CT in detecting ELST have not been established. CT is a recommended modality for preoperative planning and evaluating the extent of the tumor in relation to the anatomy of the fine temporal bone structures (2). Recent studies on the utility of 68Ga-DOTA-TOC PET/CT for diagnosing neuroendocrine tumors indicate that 68Ga-DOTATATE PET/CT has a higher detection rate for VHL-associated lesions compared to CT and MRI. Given that 68Ga-DOTATATE PET/CT has lower radiation exposure than a CT scan, it could be a reasonable alternative for screening and surveillance of VHL tumors when MRI is contraindicated or limited (23).

The cochlear implant used in this procedure was the Cochlear Nucleus CI24RE, which is conditionally compatible with MRI up to 1.5 Tesla. Recent advances in cochlear implant design now allow for compatibility with 3-Tesla MRI. Depending on the imaging protocol, the artifact can be significantly reduced in devices with rotary magnets, such as the device implanted in our patient (24). In doubtful cases, the magnet can be temporarily removed in a minimally invasive surgical procedure for the duration of the MRI (25).

Cochlear implantation in ELST patients is uncommon; however, a recent meta-analysis by Giocchini et al. estimated the pooled proportion of profound sensorineural hearing loss in ELST at 43.4% across 118 cases of the tumor (1). In case of bilateral tumors or bilateral hearing loss, cochlear implantation should be considered. If the patient or the surgeon decides against simultaneous implantation, implanting a dummy electrode is strongly advised. Subsequent cochlear fibrosis resulting from hemorrhage in the scala tympani or postsurgical trauma may render later attempts at implantation unsuccessful. In cochlear implant patients with VHL, the imaging follow-up schedule should include contrast-enhanced CT. An MRI should be considered if there is a strong suspicion of recurrence; the magnet should be removed, and a dummy magnet placed before the procedure.

## 5 Conclusions

The treatment of choice for endolymphatic sac tumors continues to be surgical intervention. This approach also allows for hearing restoration through CI. While a partial labyrinthectomy may be performed if required, simultaneous cochlear implantation or dummy electrode placement should be attempted in those instances. Contemporary cochlear implants boast compatibility with high-resolution MRI, and the artifact region can be minimized by using supplementary MRI protocols or magnet removal.

## Data Availability

The original contributions presented in the study are included in the article/Supplementary Material. Further inquiries can be directed to the corresponding author.
